# 888. Estimation of the Incidence of RSV-attributable Circulatory and Respiratory Hospital Admissions in Adults in Germany between 2015–2019, Overall and for Specific Conditions

**DOI:** 10.1093/ofid/ofad500.933

**Published:** 2023-11-27

**Authors:** Caroline Beese, Gordon Brestrich, Elizabeth Begier, Aleksandra Polkowska-Kramek, Robin Bruyndonckx, Charles Nuttens, Thao Mai Phuong Tran, Worku Biyadgie Ewnetu, Juan Luis Ramirez Agudelo, Lea Johanna Bayer, Caihua Liang, Bradford D Gessner, Gernot Rohde

**Affiliations:** Pfizer Pharma GmbH, Berlin, Berlin, Germany; Pfizer Vaccines, Berlin, Berlin, Germany; Pfizer Vaccines, Berlin, Berlin, Germany; P95, Leuven, Brabant Wallon, Belgium; P95, Leuven, Brabant Wallon, Belgium; Pfizer Inc, Paris, Ile-de- France, France; P95, Leuven, Brabant Wallon, Belgium; P95, Leuven, Brabant Wallon, Belgium; P95, Leuven, Brabant Wallon, Belgium; Pfizer Pharma GmbH, Berlin, Berlin, Germany; Pfizer Inc, Paris, Ile-de- France, France; Pfizer Biopharma Group, Collegeville, Pennsylvania; University Hospital Frankfurt, Frankfurt am Main, Hessen, Germany

## Abstract

**Background:**

Respiratory syncytial virus (RSV) can cause severe outcomes in adults, especially those with underlying risk factors. However, RSV incidence in adults is usually underestimated because of non-specific symptomatology and limited standard of care testing. We retrospectively estimated RSV-attributable incidence of hospital admissions in adults in Germany between 2015–2019.

**Methods:**

Information from inpatient visits was gathered from a Statutory Health Insurance database (approximately 3,5 million insured people). A quasi-Poisson regression model was used to estimate RSV-attributable incidence among both respiratory and circulatory hospital admissions accounting for within-season fluctuations and virus activity stratified by age groups (18–44, 45–59, 60–75, ≥75 years), risk status, and disease subgroups (5 respiratory and 4 circulatory).

**Results:**

The highest RSV-attributable incidence rates of hospital admissions were observed for circulatory diseases among older patients and those with risk factors (Table). In adults aged 60 years or older, arrythmia and acute coronary/myocardial infarction accounted for the highest incidence and stroke for the lowest incidence within circulatory hospitalizations (Table). Within respiratory hospitalizations in adults ≥75 years, RSV-attributable incidence was highest for hospital admissions related to influenza/pneumonia and chronic obstructive pulmonary disease; it was lowest for hospital admissions related to upper respiratory diseases (Table).

RSV Incidence Rate Ranges

**Figure:**
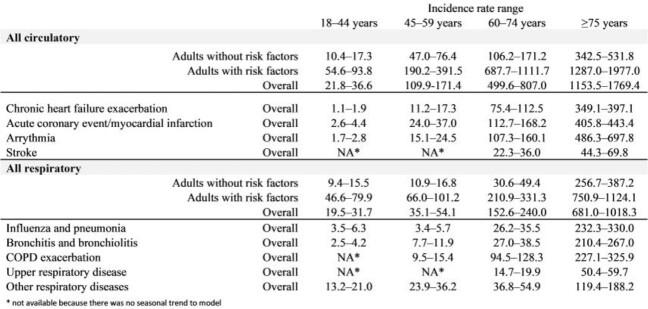

Estimated RSV-attributable Incidence Rate per 100,000 Inhabitants Related to Circulatory and Respiratory Diseases in Adults Reported as Ranges from 2015 to 2019, Stratified by Age Group (in Years) and Risk Status, Germany

**Conclusion:**

This study showed that RSV causes a considerable burden of hospitalizations in adults in Germany, and, like other respiratory viruses’ (e.g., influenza and SARS-CoV-2), contributes to both respiratory and circulatory hospitalizations. This highlights the need to implement effective prevention strategies especially among the oldest and those with underlying risk factors.

**Disclosures:**

**Caroline Beese, n/a**, Pfizer: CB is an employee of Pfizer, the sponsor of this study **Gordon Brestrich, PhD**, Pfizer: GB is an employee of Pfizer, the sponsor of this study **Elizabeth Begier, M.D., M.P.H.**, Pfizer: EB is an employee of Pfizer, the sponsor of this study|Pfizer: Stocks/Bonds **Aleksandra Polkowska-Kramek, n/a**, P95: APK is an employee of P95, paid by Pfizer to conduct the study **Robin Bruyndonckx, n/a**, P95: RB is an employee of P95, paid by Pfizer to conduct the study **Charles Nuttens, n/a**, Pfizer: CN is an employee of Pfizer, the sponsor of this study **Thao Mai Phuong Tran, n/a**, P95: TMPT is an employee of P95, paid by Pfizer to conduct the study **Worku Biyadgie Ewnetu, n/a**, P95: WBE is an employee of P95, paid by Pfizer to conduct the study **Juan Luis Ramirez Agudelo, n/a**, P95: JLRA is an employee of P95, paid by Pfizer to conduct the study **Lea Johanna Bayer, n/a**, Pfizer: LJB is an employee of Pfizer, the sponsor of this study **Caihua Liang, MD, PhD**, Pfizer: CL is an employee of Pfizer, the sponsor of this study **Bradford D. Gessner, M.D., M.P.H.**, Pfizer: I am an employee of Pfizer|Pfizer: Stocks/Bonds

